# Multifocal Cutaneous Abscesses

**DOI:** 10.1093/cid/ciag074

**Published:** 2026-07-08

**Authors:** Shrruthi Velavan, Shreya K Gowda, Devasenathipathy Kandasamy, Gomathy Sethuraman

**Affiliations:** Department of Dermatology, All India Institute of Medical Sciences, New Delhi, India; Department of Dermatology, All India Institute of Medical Sciences, New Delhi, India; Department of Radiodiagnosis and Interventional Radiology, All India Institute of Medical Sciences, New Delhi, India; Department of Dermatology, All India Institute of Medical Sciences, New Delhi, India

**Figure 1.**
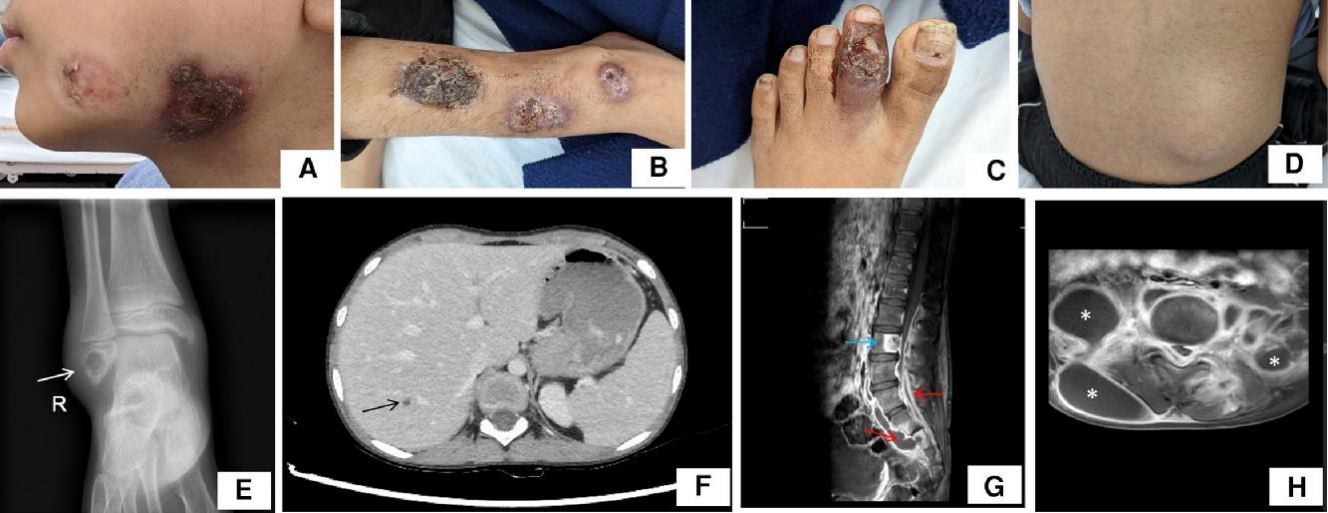
*A*, Shows diffuse swelling of the left mandibular region with overlying two erythematous, well-defined, crusted plaques discharging seropurulent exudate. *B*, Three erythematous, “boggy” plaques with crusting and purulent discharge on the right lower leg. *C*, Fusiform swelling with ulceration and crusting of the left second toe. *D*, Shows a localized, nontender, nonerythematous swelling measuring 8 × 7 cm on the right para-spinal area. *E*, Radiograph showing a lytic lesion in the right fibula. *F*, CECT abdomen revealing a hypodense (granulomatous) lesion in the liver. *G*, T1-weighted fat-suppressed postcontrast image in sagittal plane shows enhancement in L4 (blue arrow), S2, and S3 vertebral bodies. There are associated prevertebral and intradural epidural abscesses extending from L3 to S4 (red arrows). *H*, Axial plane MRI demonstrates multiple abscesses in the bilateral paravertebral region involving psoas muscles and erector spinae muscles, reaching up to the skin (*). Inflammation also extends into the epidural space.

An 11-year-old boy from Vijay Nagar, Delhi, an urban area of North India, presented with a 3-year history of progressive swelling in the left mandibular region, accompanied by overlying erythematous crusted plaques discharging seropurulent exudate. He also had erythematous “boggy” plaques with crusting and purulent discharge on the right lower leg, along with fusiform ulcerated swelling of the left second toe. His parents observed a nontender, localized swelling measuring 8 × 7 cm in the right para-spinal area 6 months prior (Figure 1*A*–*D*). Initially, he was treated as a case of recurrent skin or soft tissue infection with multiple antibiotics. Due to inadequate response and the appearance of new lesions causing diagnostic uncertainty, he was referred to a tertiary care center. On examination, bilateral submandibular, cervical, and inguinal lymphadenopathy with splenomegaly was noted. There was no similar history among family members. A workup for primary immunodeficiency was unremarkable.

His investigations revealed iron deficiency anemia, elevated ESR (erythrocyte sedimentation rate), and C-reactive protein. The radiograph showed a lytic lesion in the right fibula (Figure 1*E*). CECT (contrast-enhanced computed tomography) of the chest and abdomen (in Figure 1*F*) and contrast magnetic resonance imaging of the spine are depicted in Figure 1*G* and 1*H*.

**What is the diagnosis?**

**Diagnosis**

Multifocal scrofuloderma (SFD)/gumma with dissemination in the lungs, liver, lymph nodes, spine, and bones.

**Figure 2.**
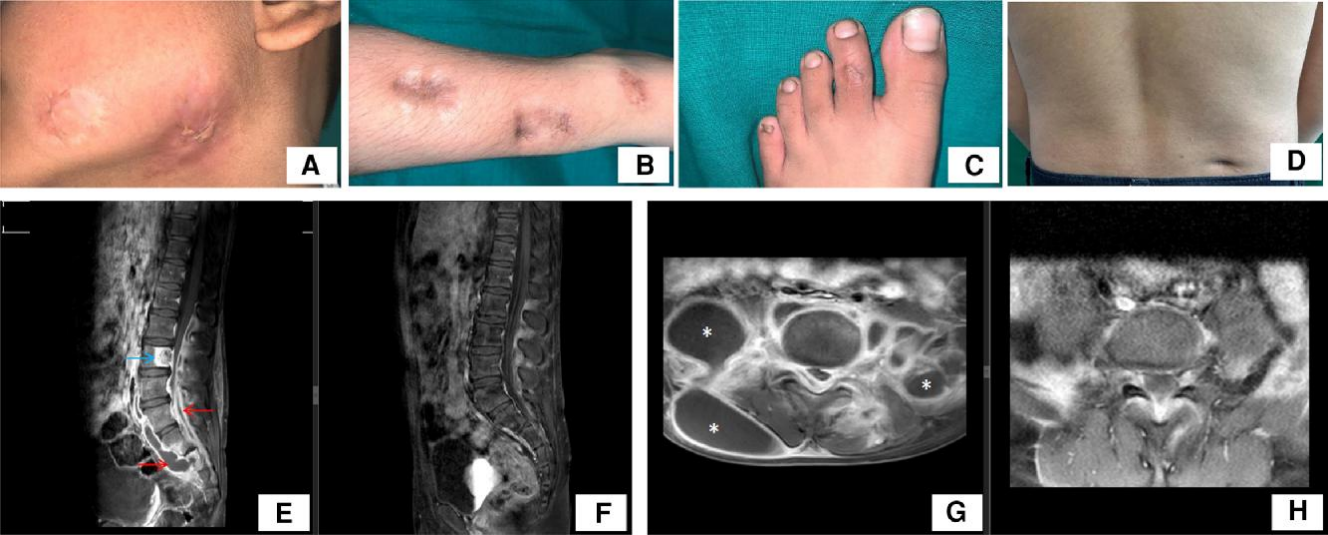
*A–D*, Post-treatment photograph showing complete healing of all the lesions. *E* and *G*, Pretreatment MRI for comparison. *F*, Post-treatment MRI shows complete resolution of bony lesions except for a thin linear enhancement in the presacral region and ill-defined enhancement posterior to the spinal canal extending from L3 to S2 (sagittal plane). *H*, Post-treatment MRI shows a significant reduction in the abscesses with residual inflammation seen in bilateral paravertebral area and subtle inflammation around the epidural space (axial plane). Abbreviation: MRI, magnetic resonance imaging.

Multiple erythematous crusted plaques with para-spinal cold abscess suggested tuberculosis. Fine-needle aspiration cytology (FNAC) revealed necrotizing granuloma with acid-fast bacilli, and culture confirmed *Mycobacterium tuberculosis*. GeneXpert MTB/RIF showed rifampicin sensitivity. Magnetic resonance imaging (MRI) supported the diagnosis. Mantoux was reactive; other tests were normal. Antituberculous therapy [ATT (ethambutol 575 mg, isoniazid 225 mg, rifampicin 375 mg, and pyrazinamide 850 mg)] was initiated, and the para-spinal abscess was drained under ultrasound guidance. Within 3 months, all skin lesions showed marked clinical improvement, and by 6 months, there was complete resolution of both the cutaneous lesions and the para-spinal abscess (Figure 2*A*–*D*). MRI was repeated after 9 months and shown in Figure 2*F* to 2*H*. Due to the multifocal nature and systemic dissemination involving bones, it was decided to continue maintenance ATT (isoniazid and rifampicin) for 18 months (author's choice).

Cutaneous tuberculosis is a rare, chronic granulomatous disease caused by *Mycobacterium tuberculosis* and occasionally by *M. bovis*. In 2024, the WHO estimated 10.8 million global tuberculosis (TB) cases, with extrapulmonary TB making up 16% [1]. In India, extrapulmonary TB accounts for 15%–24% of cases, with cutaneous TB representing 1%–1.5% [2]. The prevalence of childhood cutaneous TB in India ranges from 18% to 54% of all skin TB cases, most commonly in the 10–14-year age group. Systemic involvement has been reported in 12.7%–53.4% of cases [3].

Cutaneous tuberculosis can result from either exogenous inoculation (eg, tuberculous chancre, tuberculosis verrucosa cutis, and rarely lupus vulgaris) or endogenous spread through hematogenous (lupus vulgaris, tuberculous gumma, and miliary TB), contiguous (SFD and tuberculosis cutis orificialis), or lymphatic routes. Tuberculids are hypersensitivity reactions to *M. tuberculosis* and include lichen scrofulosorum and papulonecrotic tuberculids. The most common forms in children are SFD, lupus vulgaris, and lichen scrofulosorum. SFD is the most common form of childhood cutaneous tuberculosis in developing countries. It usually results from contiguous spread from underlying tuberculous foci in lymph nodes, bone, joints, testes, or lacrimal glands, most often cervical or inguinal nodes [4, 5]. Lesions start as painless subcutaneous cold abscesses that rupture into ulcers or sinus tracts with undermined bluish margins, healing with puckered scars. Variants include symmetrical or segmental forms and can be associated with lupus vulgaris or lichen scrofulosorum. Severe, extensive cases involving several groups of lymph nodes and multiple communicating sinus tracts are called scrofulous gumma [4]. Tuberculous gumma is a deep cutaneous form of hematogenously disseminated tuberculosis, appearing as cold, fluctuant subcutaneous nodules that eventually form abscesses. Unlike SFD, which spreads directly from an underlying focus, tuberculous gumma develops when bacillemia seeds the skin and subcutaneous tissue [5, 6].

Diagnosis of SFD depends on clinical features supported by laboratory confirmation, with organism isolation being the gold standard. Histopathology shows 64%–85% clinicopathological concordance, while FNAC helps identify SFD and associated lymph node disease. The tuberculin skin test and interferon-γ release assay serve as adjunctive tools. Children with multifocal lesions should be evaluated for systemic involvement, especially of the lungs, bones, and abdomen. In cases of disseminated disease, high bacillary load, or poor response to antitubercular therapy, drug resistance must be suspected [4]. SFD resembles several granulomatous infections such as cutaneous Leishmaniasis, disseminated sporotrichosis, atypical mycobacterial (ATM) infection, melioidosis, and brucellosis. Noduloulcerative cutaneous Leishmaniasis mimics SFD, presenting as inflammatory/edematous and erythematous papuloplaques evolving into verrucous nodules with ulceration and crusting. Histology shows pseudoepitheliomatous hyperplasia, plasma cell-rich granulomas, and Leishmania amastigotes on Giemsa stain.

Sporotrichosis appears as verrucous or ulcerated plaques; histology reveals granulomatous inflammation, Splendore–Hoeppli phenomenon, and cigar-shaped yeast on Periodic Acid Schiff/Gomori Methenamine Silver stain.

Cutaneous ATM, such as *Mycobacterium scrofulaceum*, *M. kansasii*, and the *M. avium* complex, resemble SFD both clinically and histologically. *M. scrofulaceum* causes chronic cervical lymphadenitis with ulceration and sinus formation, while *M. kansasii* and *M. avium* complex present as noduloulcerative plaques or indurated nodules with draining sinuses, after trauma or surgery. Histology shows suppurative granulomatous inflammation and neutrophilic necrosis, sparse acid-fast bacilli on Ziehl–Neelsen stain. Culture, PCR, or line probe assays are crucial for species identification and targeted therapy [7].

Melioidosis and Brucellosis are neglected tropical and zoonotic infections caused by *Burkholderia pseudomallei* and *Brucella burgdorferi*. Both present with a wide clinical spectrum, ranging from subclinical infection to acute septicemia, subacute cutaneous abscesses, and chronic multisystem disease. Culture is confirmatory, and treatment for both includes cotrimoxazole and doxycycline [8].

The management of cutaneous tuberculosis according to the National Tuberculosis Elimination Program guidelines, includes directly observed treatment short course therapy. This involves a 2-month intensive phase with—isoniazid, rifampicin, ethambutol, and pyrazinamide followed by a 4-month continuation phase with isoniazid, rifampicin, and ethambutol. However, children with bone tuberculosis or systemic dissemination may require longer antitubercular therapy for 18 to 24 months [5, 9].

In conclusion, SFD, especially extensive lesions, warrants systemic screening since up to 55% of children can have systemic tuberculosis [10]. Early recognition and treatment of SFD/gumma are crucial in children to prevent systemic spread, disfigurement, and morbidity. In our case, the source of infection remained unidentified due to the lack of any significant contact or family history, and Mendelian susceptibility to mycobacterial disease panel could not be performed.
